# Supplementary material for: DegQ and SwrA differentially modulate protease production and flagellar motility in Bacillus subtilis
Source: bioRxiv. 2026 Oct 28:2026.10.26.762088. Preprint. [Version 1] doi: 10.64898/2026.10.26.762088 (PMC13733949; doi:10.64898/2026.10.26.762088)
Supplement: Supplementary file 1 [file NIHPP2026.10.26.762088V1-supplement-1.pdf]

bioRxiv preprint doi: https://doi.org/10.64898/2026.10.26.762088; this version posted October 28, 2026. The copyright holder for this preprint (which was not certified by peer review) is the author/funder, who has granted bioRxiv a license to display the preprint in perpetuity. It is made available under a CC-BY 4.0 International license.

Table S1

Plasmid	Genotype
pDP639	*amyE::*P_{aprE}*-lacZ cat amp*
pAH25	*amyE::spec amp*

bioRxiv preprint doi: https://doi.org/10.64898/2026.10.26.762088; this version posted October 28, 2026. The copyright holder for this preprint (which was not certified by peer review) is the author/funder, who has granted bioRxiv a license to display the preprint in perpetuity. It is made available under a CC-BY 4.0 International license.

pAC001	*ΩΔdegS* ori^{TsBs} *mls amp*
pAC016	*thrC::*$P_{hyspank}$*-degQ mls amp*
pAEJ15	*amyE::*P_{degQ}*-lacZ cat amp*
pDG1515	*tet amp*
pDG268	*amyE::lacZ cat amp*
pDP150	*thrC::*$P_{hyspank}$ *mls amp*
pDP486	*amyE::*P_{degS}*-degS spec amp*
pDP487	*amyE::*P_{degS}*-degU spec amp*
pDR111	*amyE::*$P_{hyspank}$ *spec amp*
pEC20	*amyE::*$P_{hyspank}$*-degQ spec amp*
pMiniMAD	ori^{TsBs} *mls amp*

Table S2

Primer	Sequence
2957	AGGAGAAGCTTGTTCACCATACACAATTCATTGATCT
2958	CTCCTGCTAGCATCCATTTATTGTATCGGTAGAACGA
3250	ACGACTCACTATAGGGCGAATTG
3251	CTCACTAAAGGGAACAAAAGCTGG
5170	GAAATTATGAATAAAACAATAGATTCGAAAATAGGTCTTGGGACATTTATTATGAT
5171	AGGAGGGTACCCCACAACGGCTTGCGTCCG
5172	AGGAGGGATCCGTCACTGTCAATTCAGAAAAAGCG
5173	TTATCGAATCTATTGTTTTATTCATAATTTCCCTCCGTCACGGCG
5299	AGGAGGTCGACGCTGACCCTCCTGCTAAGC
5300	CTCCTGGATCCTGCTTTATAACTCTATTGTAAGG
5301	CTCCTGGATCCCTTGTATTATTGTTCTATATACTC
5310	AGGAGGCTAGCACCTAGGACTCGTTCACCATACACAATTC
5311	AGGAGGCATGCACGAAAAAAAAGACTTGTTTCCAAGTC
8119	AGGAGGAATTCGATAAAGGTATCAATATTGTTGGCAGG
8120	CTCCTGGATCCGAAAGATCAATGAATTGTGTATGGTG
8442	AGGAGGAATTCCCTTGCAAATCGGATGCCTG
8443	CTCCTGGATCCCAGAGTAGACTTACTTAAAAGAC
8506	GTAGAGACCCGAAAAGAATATAAACAG
8507	CAATTCGCCCTATAGTGAGTCGTCAAGTTCGAGTCGGAATAACAATTGTT
8508	CCAGCTTTTGTTCCCTTTAGTGAGCAAAAGCATTGATCAACTCGATAAAT
8509	CAGACTGTTAATATGACTCAAATATCC
